# Supplementary material for: Evidence that polyploidy in esophageal adenocarcinoma originates from mitotic slippage caused by defective chromosome attachments
Source: Cell Death Differ. 2021 Mar 1;28(7):2179–93. doi: 10.1038/s41418-021-00745-8 (PMC8257792; doi:10.1038/s41418-021-00745-8)
Supplement: Supplementary file 1 — Supplementary Information [file 41418_2021_745_MOESM1_ESM.docx]

Supplementary Information for:

**Evidence that polyploidy in esophageal adenocarcinoma originates from mitotic slippage caused by defective chromosome attachments**

Stacey J. Scott^1^, Xiaodun Li^2^, Sriganesh Jammula^3^, Ginny Devonshire^3^, Catherine Lindon^4^, Rebecca C. Fitzgerald^2^ and Pier Paolo D’Avino^1*^

^1^Department of Pathology, University of Cambridge, Tennis Court Road, Cambridge, CB2 1QP, UK

^2^Medical Research Council Cancer Unit, Hutchison/Medical Research Council Research Centre, University of Cambridge, Cambridge, UK

^3^Cancer Research UK Cambridge Institute, University of Cambridge, Li Ka Shing Centre, Cambridge, UK

^4^Department of Pharmacology, University of Cambridge, Tennis Court Road, Cambridge, CB2 1PD, UK

**This PDF file includes:**

Legends for Supplementary Figures S1 to S4

Supplementary Tables S1 and S4

Legends for Supplementary Tables S2, S3 and S5

Legends for Supplementary Videos S1 to S4

**Other Supplementary Information for this manuscript include the following:**

Videos S1 to S4

Supplementary Table S2, S3, and S5

**Supplementary Figure Legends**

**Figure S1. BE and EAC cells displays specific mitotic defects.** Graph showing the percentages of BE and OAC cells showing one of three phenotypes: lagging chromatin, multipolar spindles or scattered chromosomes. Bars indicated SEM. More than 3000 cells in total and more than 200 mitotic cells per each cell line were counted; n≥6 independent experiments.

**Figure S2. EAC cells display extra centrosomes.** (**a**) Representative images from the indicated BE and EAC cell lines fixed and stained to detect Plk4 (red in the merged images), γ-tubulin (green in the merged images) and DNA (blue in the merged images). Bars, 10 μm. (**b-c**) Graphs showing the quantification of the number of centrosomes (B) and the defects (C) observed in in the mitotic cells from the experiments in (A). At least 50 mitotic figures from at least 3 different experiments were analyzed for each cell line. Bars indicated SEM; **** *p*<0.0001 (Two-way ANOVA with multiple comparisons).

**Figure S3. BE and EAC cells have a functional spindle assembly checkpoint**. (**a**) Indicated BE and EAC cells were treated with the microtubule depolymerizing drug nocodazole or its solvent DMSO for 18 hours and then fixed and stained to detect the mitotic marker histone H3 pS10 (red in the merged images), tubulin (green in the merged images) and DNA (blue in the merged images). Bars, 10 μm. (**b**) Graph showing the quantification of cells in mitosis (mitotic indices; MI) for each cell line from the experiment described in (A). (**c**) Graph showing the MI fold increase for each cell line from the experiment described in (A). More than 1500 cells were counted for each cell line; n≥3 independent experiments.

**Figure S4. EAC organoids.** Phase contrast images of the organoids used in the experiments shown in Fig. 3. Bars, 50 μm.

**Table S1. List of cell lines used in our experiments.**

| Cell line | Origin | Gender | Age | Ploidy | p53 | Reference  /source |
| --- | --- | --- | --- | --- | --- | --- |
| RPE1 | Retinal epithelium, h-TERT immortalised | Female | Adult | Diploid | Wild type | [1]/ATCC |
| CPA | Non dysplastic BE, h-TERT immortalised | Male | Adult | Near diploid | Wild type | [2]/ATTC |
| CPD | Dysplastic BE, h-TERT immortalised | Male | Adult | Near tetraploid | c.404G>A | [2]/ATTC |
| FLO | EAC | Male | 68 | Near tetraploid | c.830G>T | [3]/EACC |
| JH-Eso-AD1 | EAC stage 3, moderate to poor differentiation | Male | 66 | Near tetraploid | c.797G>A | [4]/to be deposited to ATCC |
| OE19 | EAC stage 2, moderate differentiation | Male | 72 | Near tetraploid | c.929dup | [5]/EACC |
| OE33 | EAC stage 2, poor differentiation | Female | 73 | Near tetraploid | c.404G>A | [5]/EACC |

**Table S4. List of organoids used in our analyses.**

| IDs | Age | Gender | Differentiation | p53 status | Ploidy |
| --- | --- | --- | --- | --- | --- |
| NG088 | 76 | Male | Moderate | Wild type | Not known, but likely diploid |
| CAM277 | 80 | Female | Poor | c.414delG, loss of p53 expression at protein level | Tetraploid genome, 100% of metaphases have a ploidy of >55. Ploidy of 4.04 indicating this organoid has experienced WGD. |
| CAM401 | 77 | Female | Poor | Over-expressed p53 with hotspot mutation (R175H) | Aneuploid genome, ploidy of 1.74. Analysis of metaphases showed the following ploidy results: ~ 90% <40, ~5% 41-45 and ~5% 47-55. |
| CAM408 | 60 | Male | Moderate | c.586G>A, loss of p53 expression at protein level | Aneuploid genome, analysis of metaphases showed the following ploidy results: ~10% <40, ~70% 41-45, ~5% 46 and ~5% >55. Overall ploidy score 1.94 |
| CAM486 | 72 | Male | Moderate | Over-expressed p53 with mutation C.731C>T | Not determined |
| CAM423 | 55 | Male | Moderate to poor | Wild type *TP53* and p53 expression pattern | Tetraploid genome. Ploidy 4.48 indicating this organoid has experienced WGD |

NG088 is the control organoid derived from non-cancerous cells from the stomach, the other organoids are all derived from EACs. All cultures are mixed heterogenous populations composed of cells with a variety of changes in chromosome number as well as chromosome rearrangements [6].

**Other Supplementary Table Legends**

**Supplementary Table S2.**

Two-way ANOVA statistical analysis of the data relative to the graph shown in Fig. 1B. Multiple comparisons are shown in a separate sheet.

**Supplementary Table S3.**

Two-way ANOVA statistical analysis of the data relative to the graph shown in Fig. 1E. Multiple comparisons are shown in a separate sheet.

**Supplementary Table S5.**

List of the genes involved in chromosome attachment selected for WGS and RNAseq analyses. For each gene is shown the primary gene name, entry UNIPROT ID, Ensembl ID, protein name and GO annotations.

**Supplementary Movie Captions**

Supplementary Movie S1.

This movie shows chromosome dynamics, visualized using SiR-DNA, in a CPA cell. All sequences were captured at 5 min intervals. Playback rate is 5 frames per second (FPS).

Supplementary Movie S2.

This movie shows chromosome dynamics, visualized using SiR-DNA, in a FLO cell. This cell completed mitosis, but showed lagging chromatin. All sequences were captured at 5 min intervals. Playback rate is 5 frames per second (FPS).

Supplementary Movie S3.

This movie shows chromosome dynamics, visualized using SiR-DNA, in a FLO cell. This cell failed to complete mitosis after an initial attempt to enter anaphase, and then chromosomes started to drift and decondense, indicating mitotic slippage. All sequences were captured at 5 min intervals. Playback rate is 5 frames per second (FPS).

Supplementary Movie S4.

This movie shows chromosome dynamics, visualized using SiR-DNA, in a JH-Eso-AD1 cell. This cell failed to complete mitosis after an initial attempt to enter anaphase, and then chromosomes started to drift and decondense, indicating mitotic slippage. All sequences were captured at 5 min intervals. Playback rate is 5 frames per second (FPS).

**References**

1. Bodnar AG, Ouellette M, Frolkis M, Holt SE, Chiu CP, Morin GB, et al. Extension of life-span by introduction of telomerase into normal human cells. Science. 1998;279(5349):349-52.

2. Palanca-Wessels MC, Barrett MT, Galipeau PC, Rohrer KL, Reid BJ, Rabinovitch PS. Genetic analysis of long-term Barrett's esophagus epithelial cultures exhibiting cytogenetic and ploidy abnormalities. Gastroenterology. 1998;114(2):295-304.

3. Hughes SJ, Nambu Y, Soldes OS, Hamstra D, Rehemtulla A, Iannettoni MD, et al. Fas/APO-1 (CD95) is not translocated to the cell membrane in esophageal adenocarcinoma. Cancer Res. 1997;57(24):5571-8.

4. Alvarez H, Koorstra JB, Hong SM, Boonstra JJ, Dinjens WN, Foratiere AA, et al. Establishment and characterization of a bona fide Barrett esophagus-associated adenocarcinoma cell line. Cancer Biol Ther. 2008;7(11):1753-5.

5. Rockett JC, Larkin K, Darnton SJ, Morris AG, Matthews HR. Five newly established oesophageal carcinoma cell lines: phenotypic and immunological characterization. Br J Cancer. 1997;75(2):258-63.

6. Li X, Francies HE, Secrier M, Perner J, Miremadi A, Galeano-Dalmau N, et al. Organoid cultures recapitulate esophageal adenocarcinoma heterogeneity providing a model for clonality studies and precision therapeutics. Nature communications. 2018;9(1):2983.
