# Supplementary figures and images for: Evidence that polyploidy in esophageal adenocarcinoma originates from mitotic slippage caused by defective chromosome attachments
Source: Cell Death Differ. 2021 Mar 1;28(7):2179–93. doi: 10.1038/s41418-021-00745-8 (PMC8257792; doi:10.1038/s41418-021-00745-8)

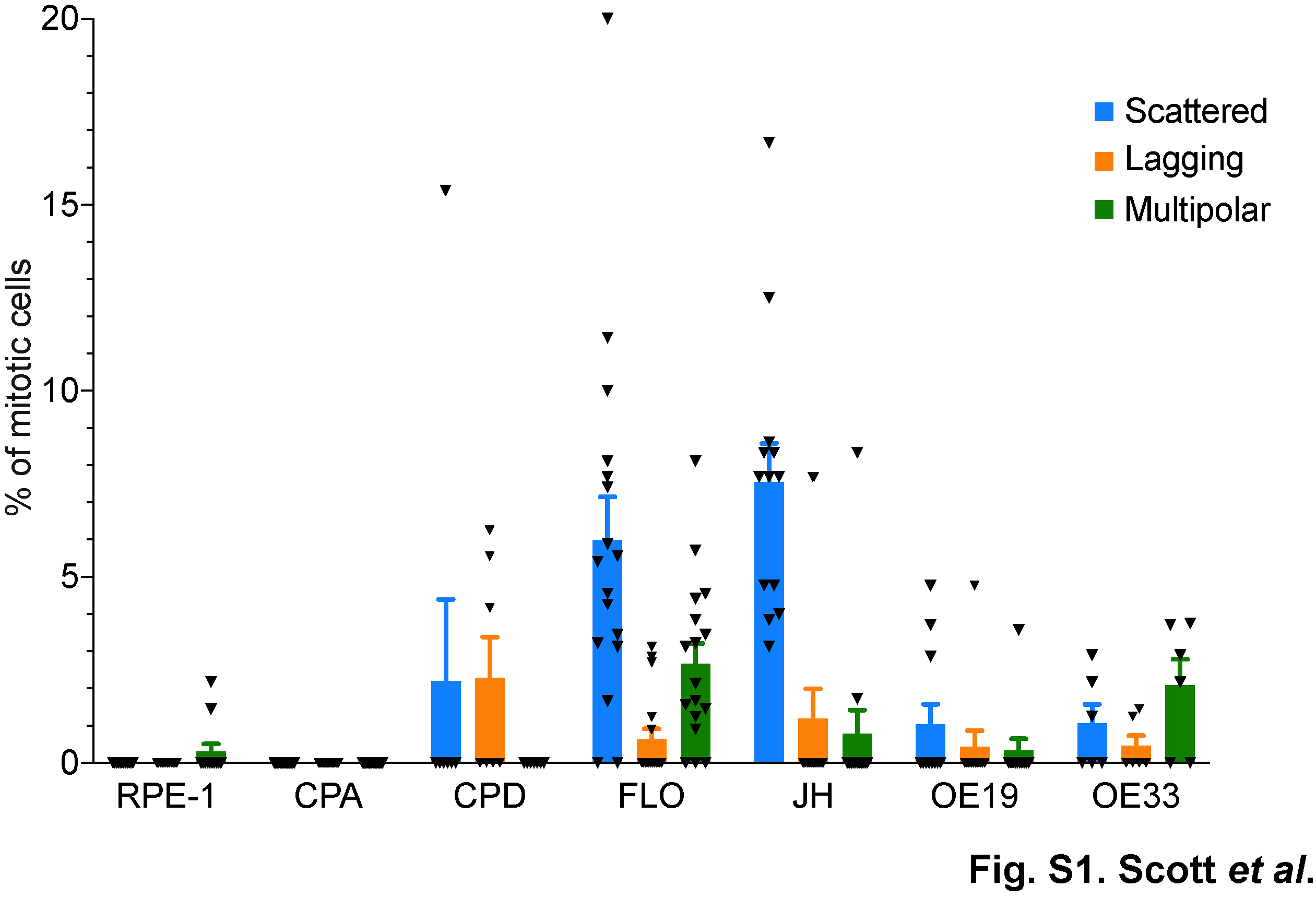

Supplement: Supplementary file 2 — Supplementary Figure S1 [file 41418_2021_745_MOESM2_ESM.tif]

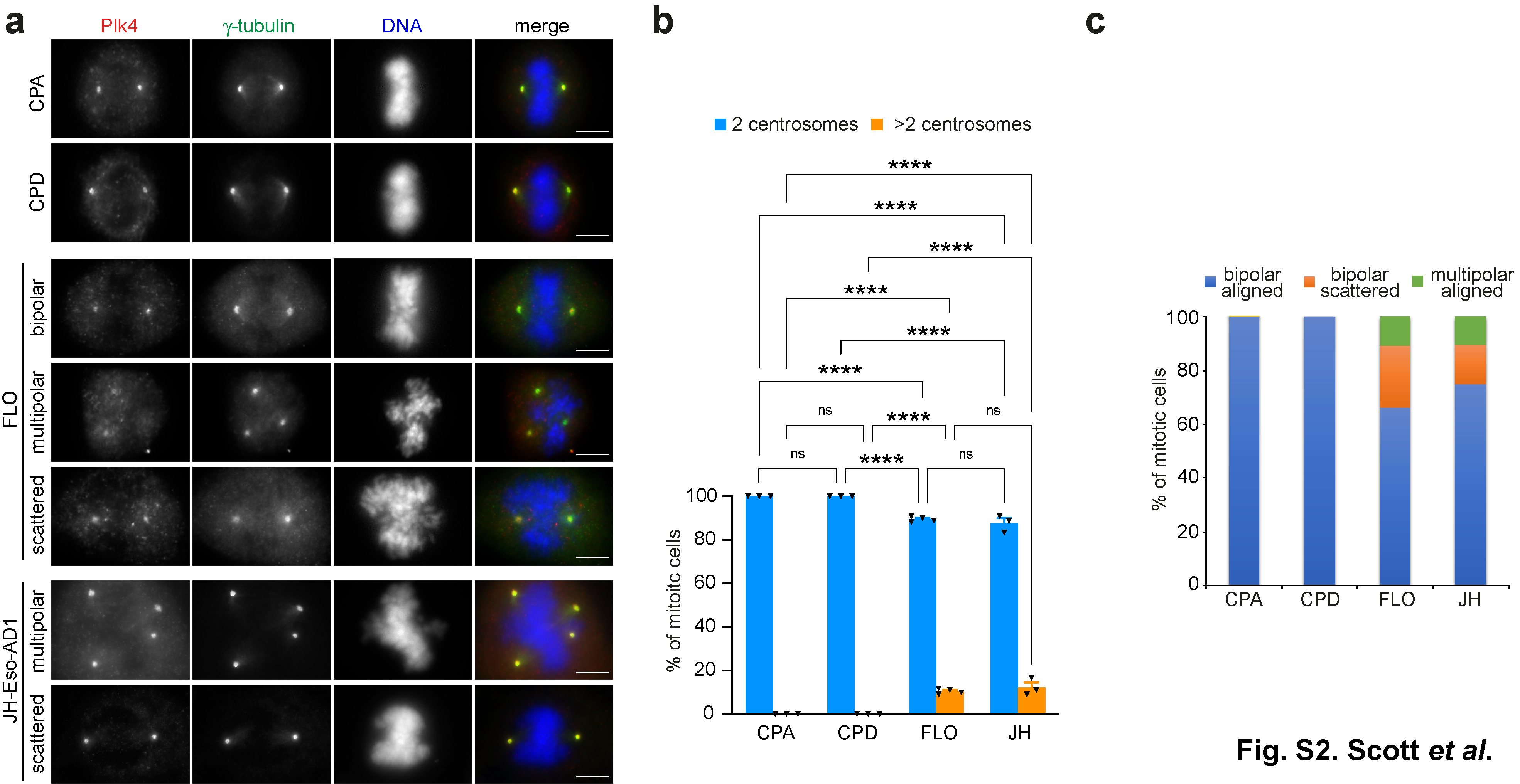

Supplement: Supplementary file 3 — Supplementary Figure S2 [file 41418_2021_745_MOESM3_ESM.tif]

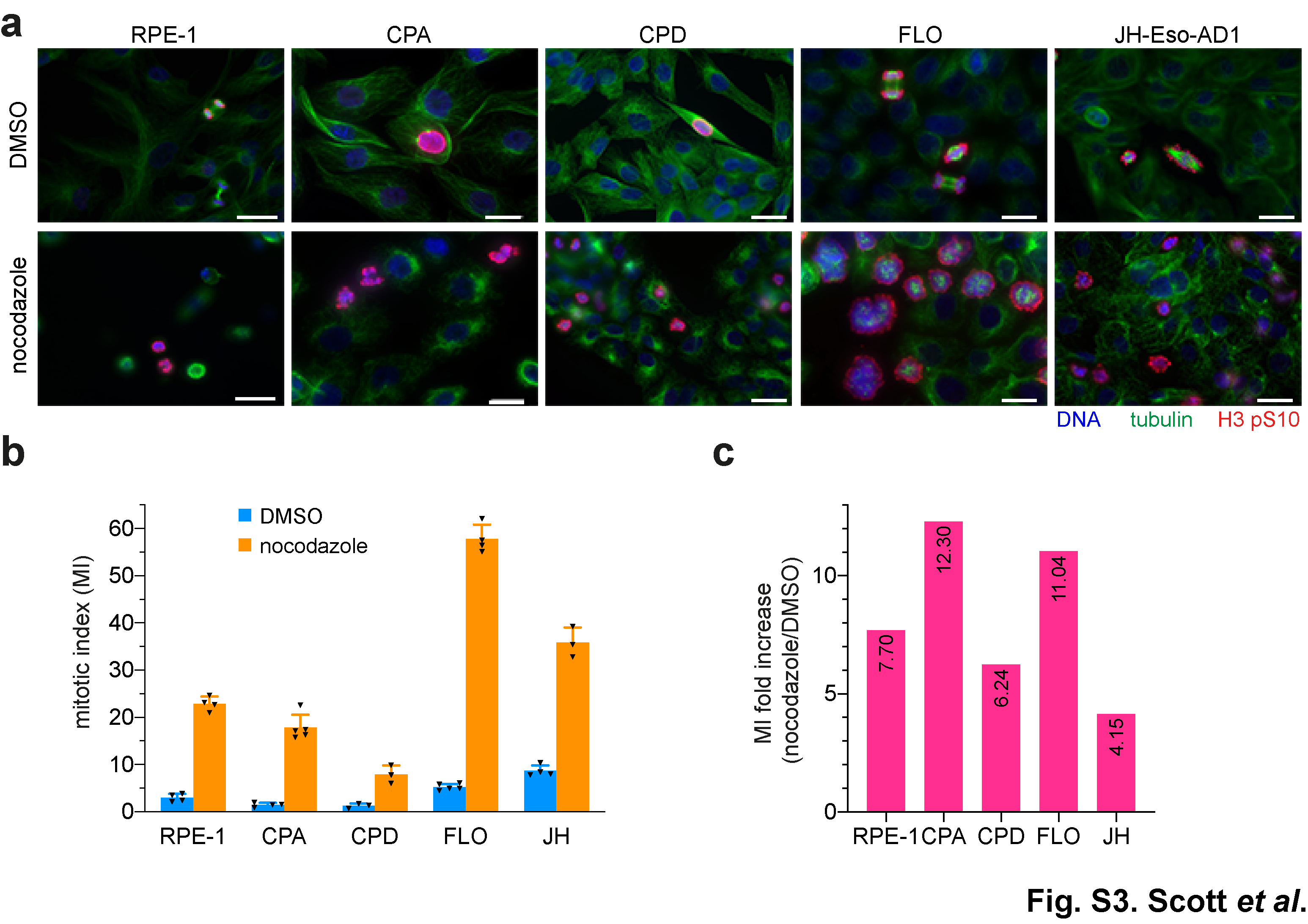

Supplement: Supplementary file 4 — Supplementary Figure S3 [file 41418_2021_745_MOESM4_ESM.tif]

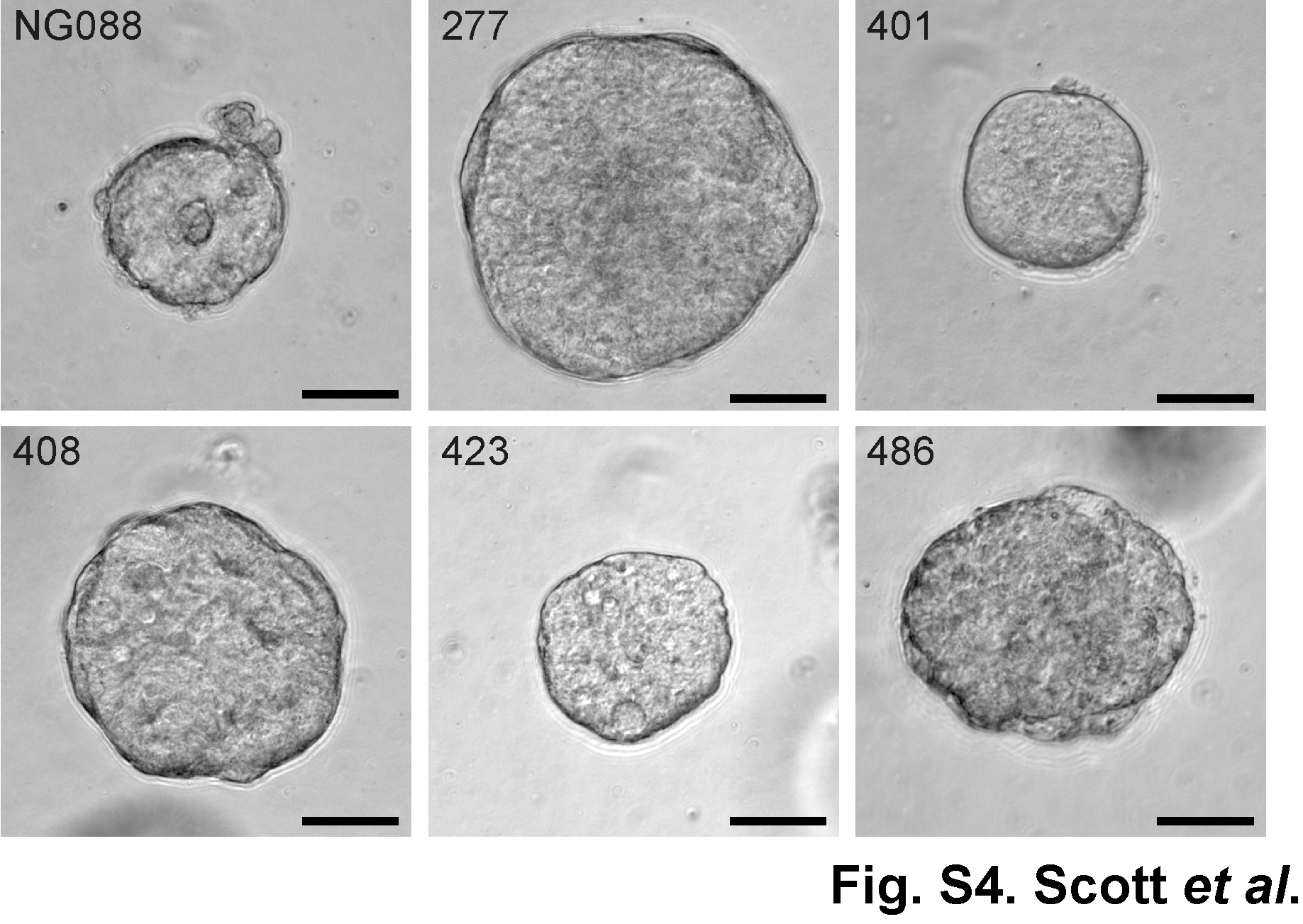

Supplement: Supplementary file 5 — Supplementary Figure S4 [file 41418_2021_745_MOESM5_ESM.tif]
